# Risk factor for interstitial pregnancy following ipsilateral salpingectomy? A retrospective matched case control study

**DOI:** 10.1186/s12884-023-06132-0

**Published:** 2023-11-30

**Authors:** Wei-Fang Wu, Jing-Song Yi, Xi Xie, Chao-Bin Liu

**Affiliations:** Department of Obstetrics and Gynecology, Fujian Maternity and Child Health Hospital, College of Clinical Medicine for Obstetrics & Gynecology and Pediatrics, Fujian Medical University, 18, Daoshan Rd, 350001 Fuzhou, Fujian China

**Keywords:** Interstitial pregnancy, Salpingectomy, Hydrosalpinx, Propensity score matching, Risk factor

## Abstract

**Background:**

Interstitial pregnancy may still happen even after ipsilateral salpingectomy, resulting in massive hemorrhage. Therefore, the purpose of the study is to identify risk factors associated with interstitial pregnancy following ipsilateral salpingectomy and discuss possible prevention.

**Methods:**

We conducted a retrospective cohort study in a single, large, university-affiliated hospital. Data of 29 patients diagnosed with interstitial pregnancy following ipsilateral salpingectomy from January 2011 to November 2020 were assigned into the case group (IP group). Whereas there were 6151 patients with intrauterine pregnancy after unilateral salpingectomy in the same period. A sample size of 87 control patients was calculated to achieve statistical power (99.9%) and an α of 0.05. The age, BMI and previous salpingectomy side between the two group were adjusted with PSM at a ratio of 1:3. After PSM, 87 intrauterine pregnancy patients were successfully matched to 29 IP patients.

**Results:**

After PSM, parous women were more common and intrauterine operation was more frequent in the IP group compared with control group (P<0.05). There was only one patient undergoing IVF-ET in the IP group as compared with 29 cases in the control group (3.4% vs. 33.3%, P<0.05). Salpingectomy was performed on 5 patients in the IP group and 4 patients in the control group due to hydrosalpinx (P<0.05). Logistic regression indicated that hydrosalpinx was the high risk factor of interstitial pregnancy following ipsilateral salpingectomy (OR = 8.175).

**Conclusions:**

Hydrosalpinx appears to be an independent factor contributing to interstitial pregnancy following ipsilateral salpingectomy in subsequent pregnancy.

## Background

Interstitial pregnancy (**IP**) refers to implantation of an embryo in the proximal fallopian tube passing through the myometrium. Its incidence ranges from 2 to 4% among all tubal pregnancies [1]. Salpingectomy is the most frequently performed procedure for tubal pregnancy to avoid recurrence of tubal pregnancy at the same side [2].It is also universally recommended in case of evident hydrosalpinx in infertile women scheduled for assisted reproductive technologies [3].It was originally thought that the risk of an ectopic pregnancy at the same site could be eliminated by removing the fallopian tube [4].However, even if the whole visible length of the tube is excised, there still remains the interstitial part [5].Thus ectopic pregnancy in the interstitial portion of tube after ipsilateral salpingectomy may still happen.

The occurrence of interstitial ectopic pregnancy at the site of a previous salpingectomy is extremely rare, approximately accounting for 0.4–1.16% of all ectopic pregnancies [6, 7]. While a rare phenomenon, the mortality rate of interstitial pregnancy is considerably 7 times higher than tubal pregnancy occurring in other segments [8].Previous observational study [9] have reported that ipsilateral salpingectomy, previous ectopic pregnancy, and in vitro fertilization are predisposing factors for interstitial pregnancy. However, the mechanisms of recurrent ipsilateral ectopic pregnancy are mainly based on clinical case reports, and no definitive data from randomized trials are available on this topic. Therefore, the aim of the present study was to assess the risk factors of interstitial pregnancy following ipsilateral salpingectomy with the hope of providing advice on prevention .

## Methods

### Participants and procedure

This retrospective comparative study was conducted using data from a single, large university-affiliated center. All pregnancies with a history of ipsilateral salpingectomy total or isthmic partial between January 1, 2011 and November 31, 2020 were reviewed. The Ethics Committee approved the study.

The diagnosis of interstitial pregnancy was made by transvaginal sonography when the following criteria were met: absence of the gestational sac in the uterine cavity, presence of an interstitial line sign (an echogenic line in the cornual region of the uterus bordering the mid-portion of the gestational sac), and a thin myometrial layer (5 mm) surrounding the gestational sac [10]. Patients were assigned to the IP group, if they were diagnosed with interstitial pregnancy at the same side of salpingectomy. All other patients were assigned to the control group, if the pregnancy was intrauterine pregnancy. For all patients with history of bilateral salpingectomy or cornual resection were excluded.

For both cases and controls, clinical data and demographic data were extracted from the electronic health record, including age, body mass index, gravidity, parity, obstetric and gynecological history, detail surgical history of the previous ipsilateral salpingectomy, interval time from salpingectomy to current pregnancy. It was also documented whether the current pregnancy had been conceived naturally or by in vitro fertilization and embryo transfer (**IVF-ET**).

We calculated that a sample size of 87 control patients was needed for the study to achieve a statistical power (99.9%) and an α of 0.05. However ,as the low incidence of interstitial pregnancy following ipsilateral salpingectomy, the controls were more than 100 times larger than cases. Propensity score macthing (**PSM**) is a commonly used statistical method in research that accomplishes the removal of confounding bias from observational cohorts where the benefit of randomization is not possible [11]. The PSM was applied to choose 87 control patients with less bias. The MatchIt package in R was used to generate the propensity score-matched with a nearest neighbor matching algorithm at a ratio of 1:3. The age, BMI and previous salpingectomy side between the two group were adjusted with PSM analysis to match 87 control patients. After PSM, the preliminary related factors of interstitial pregnancy following ipsilateral salpingectomy were evaluated by univariate regression analysis with the significant parameters further analyzed by multivariate binary logistic regression analysis for determination of the influencing factors.

### Statistical analysis

Continuous variables that followed a normal distribution pattern and had homogenous variance were expressed as means ± standard deviations and were compared using Student’s t-test. Non-normally distributed data were expressed as medians and analyzed using the Mann-Whitney U test. Intergroup differences in categorical variables were compared using the chi-square or Fisher tests. In addition, a p value of < 0.05 was used in the univariate analysis for inclusion of putative risk factors. Multivariate binary logistic regression analysis was used to evaluate risk factors. Data processing and statistical analyses were completed using SPSS version 25.0 (IBM, Armonk, NY, USA) and the R software, version 3.3.1 (R Foundation for Statistical Computing, Vienna, Austria). All p-values reported are 2 sided and a *p* < 0.05 was considered statistically significant.

## Results

During the study period, there were 8877 ectopic pregnancies in our institution. Among them, 270 patients were interstitial pregnancy, of which 29 (10.7%,29/270) had the history of homolateral salpingectomy, 18(6.7%, 18/270) had the history of contralateral salpingectomy, and 9 (3.3%, 9/270) had the history of bilateral salpingectomy. The incidence of interstitial pregnancy following ipsilateral salpingectomy was 0.33% in all ectopic pregnancies. Whereas there were 6151 patients with intrauterine pregnancy after unilateral salpingectomy in the same period.

These 29 patients with history of homolateral salpingectomy were assigned into the case group (IP group). The age of the IP group ranged from 20 to 38 years old, with an average of 29.6 ± 4.52 years. BMI of patients in IP group was 20.8 ± 3.2 kg/m^2^.The number of previous gravidity varied from 0 to 7, and the number of previous deliveries varied from 0 to 3. Women with a history of previous intrauterine operation were 21.The cases of previous salpingectomy performed on right side was 22 (75.9%), while 7 (24.1%) on the left side. Salpingectomy was performed in 23 cases owing to ectopic pregnancy, 5 cases owing to hydrosalpinx and 1 case owing to ovarian torsion. Only one patient conceived by IVF-ET. Seven patients suffered from interstitial pregnancy rupture. Twenty-seven patients were treated surgically and the surgical treatment confirmed that the pregnancy was located lateral to the round ligament in the uterotubal junction [12],whereas 2 patients received conservative management with methotrexate. Cornual resection was used to treat 23 patients and cornuostomy was used to treat 4 patients. One patient suffered from persistent ectopic pregnancy after surgery.

In the control group with 6151 intrauterine pregnancy patients, the age ranged from 20 to 44 years old, with an average of 30.2 ± 1.1 years. BMI of patients was 21.2 ± 1.8 kg/m^2^. The cases of previous salpingectomy performed on right side was 3187 (51.8%), while 2964 (48.2%) on the left side. Salpingectomy was performed in 244 (5%) cases owing to hydrosalpinx. Patients conceived by IVF-ET were 2083 (33.9%).

The age, BMI and previous salpingectomy side between the two group were adjusted with PSM at a ratio of 1:3. After PSM, 87 intrauterine pregnancy patients were successfully matched to 29 IP patients. Table 1 presents the patient demographic details before and after matching.

**Table 1:** Demographic details of two groups before and after propensity score matching (PSM)

	Before PSM			After PSM		
	IP group	Control group	*p*-value	IP group	Control group	*p*-value
**No. of patients**	29	6151		29	87	
**Mean age, year**	29.6 ± 4.5	30.2 ± 1.1	0.291	29.6 ± 4.5	30.1 ± 4.1	0.601
**BMI, kg/m**	20.8 ± 3.2	21.2 ± 1.8	0.507	20.8 ± 3.2	20.9 ± 2.7	0.869
**Salpingectomy, n (%)**						
**Left**	7(24.1)	2964(48.2)	0.010	7(24.1)	31(35.6)	0.253
**Right**	22(75.9)	3187(51.8)		22(75.9)	56(64.4)	

Note: BMI = body mass index

As shown in Table 2, after PSM, parous women were more common and intrauterine operation was more frequent in the IP group compared with control group (*P*<0.05). There was only one patient undergoing IVF-ET in the IP group as compared with 29 cases in the control group (3.4% vs. 33.3%, *P*<0.05). Salpingectomy was performed on 5 patients in the IP group as compared to 4 patients in the control group due to hydrosalpinx (17.2% vs. 4.6%, *P*<0.05).

**Table 2:** Comparison of the clinical characteristics between two group after propensity score matching

Variables	IP group(n = 29)	Control group(n = 87)	*p* Value
**Gravidity**	2.4 ± 1.7	2.0 ± 1.1	0.170
**Parity, n (%)**			
** nulliparous**	14(48.3%)	60(69.0)	0.045
** parous**	15(51.7)	27(31.0)	
**Reason for salpingectomy, n (%)**			
** Hydrosalpinx**	5(17.2)	4(4.6)	0.028
** others**	24(82.8)	83(95.4)	
**Salpingectomy-subsequent pregnancy interval**			
** ≤ 6 months**	3(10.3)	10(11.5)	0.865
** >6 months**	26(89.7)	77(88.5)	
**Previous surgical procedure, n (%)**			
** Laparoscopy**	21(72.4)	59(67.8)	0.643
** Laparotomy**	8(27.6)	28(32.2)	
**Mode of conception, n (%)**			
** spontaneous**	28(96.6)	58(66.7)	0.001
** IVF-ET**	1(3.4)	29(33.3)	
**Previous abdominal surgery ,n (%)**	10(34.5)	22(27.6)	0.337
**Previous intrauterine operation, n (%)**	21(72.4)	44 (50.6)	0.040
**Sterility ,n (%)**	6(20.7)	14(16.1)	0.570
**History of ectopic pregnancy ,n (%)**	2(6.9)	7(8.0)	0.841

Note: ET = embryo transfer; IVF = in vitro fertilization

Four parameters with significant differences in univariate analysis, i.e., parity, intrauterine operation, hydrosalpinx and mode of conception, were further analyzed by multivariate binary Logistic regression. The results indicated that the high risk factor for interstitial pregnancy following ipsilateral salpingectomy was hydrosalpinx (OR = 8.175, 95%CI = 1.323–50.519). The result of logistic regression analysis is shown in Table 3.

**Table 3:** Logistic regression analysis of risk factors for interstitial pregnancy following ipsilateral salpingectomy

Variables	OR Value	95%(CI)	*p* Value
**Parity**	2.221	0.837~5.890	0.109
**Intrauterine operation**	1.509	0.569~4.002	0.408
**Hydrosalpinx**	8.175	1.323~50.519	0.024
**IVF-ET**	0.054	0.006~0.508	0.011

Note: ET = embryo transfer; IVF = in vitro fertilization

## Discussion

Interstitial pregnancy following ipsilateral salpingectomy has been sporadically described primarily in case reports and small case series [6, 7, 12–17]. To the best of our knowledge, this is the first study assessing the risk factors of interstitial pregnancy following ipsilateral salpingectomy in a propensity-matched series. The use of PSM allows us to assess a heterogenous group of patients with less bias. In this retrospective, matched, cohort study, we find that hydrosalpinx affects the rate of interstitial pregnancy following ipsilateral salpingectomy. The mechanism by which an ectopic pregnancy locates in the remnant tube after homolateral salpingectomy has still not been completely elucidated. One possible explanation is that an oocyte may have been normally fertilized in the normal contralateral fallopian tube and later the fertilized egg may be taken up to the remnant tube via intrauterine transmigration [14, 15, 18]. The other is the external chemotactic theory, which postulates that the fertilized egg may migrate transperitoneally from the serosa into the interstitial portion of the tube before local embryonic nidation took place [6, 13].

Hydrosalpinx affects pregnancy outcomes of IVF-ET [19, 20],and thus salpingectomy is the preferred pretreatment for hydrosalpinx to improve pregnancy outcomes before IVF-ET [3, 21–23]. Our results show that there is a strong association between hydrosalpinx and interstitial pregnancy after homolateral salpingectomy. In the study of Hunter, he found that inflammation may affect normal embryo implantation [24] .However, as our study only focused on interstitial pregnancy following unilateral salpingectomy, the absolute cases of interstitial pregnancy following prior salpingectomy owing to hydrosalpinx were low. We speculate that pretreatment for hydrosalpinx before IVF-ET commonly may prefer to bilateral salpingectomy rather than unilateral salpingectomy. Secondly, even if one fallopian tube with less hydrosalpinx is preserved after operation owing to tubal infertility, salpingectomy may be performed on remnant tube owing to recurrent hydrosalpinx before IVF-ET. Therefore, whether hydrosalpinx is also a significant risk factor for interstitial pregnancy after bilateral salpingectomy requires further exploration.

There is one unexpected finding in our study. The incidence of interstitial pregnancy after IVF-ET with ipsilateral salpingectomy appears to be much lower than previous reports [9, 17]. The earlier report of 25.9% (29/121) of all ectopic pregnancies [17] related to a mixture of bilateral salpingectomy and ipsilateral salpingectomy. The exact reason is unclear. We speculate that if only one fallopian tube is resected, when fallopian tube transmigration after IVF-ET happen, the embryos may more likely move to the reserved tube with less resistance. If the remnant tube is normal, the embryos may be sent back to the uterine. Otherwise, if the remnant tube suffers with inflammation, the embryos may fertilize in the remnant tube. However, the possibility of spontaneous pregnancy after bilateral salpingectomy is tiny, and IVF-ET is a vital way to make the possibility of pregnancy after bilateral salpingectomy come true. Therefore, either intrauterine or ectopic pregnancy after bilateral salpingectomy mainly occurs after IVF-ET.

The current study failed to show differences in the interval from salpingectomy to subsequent pregnancy. However, in a study by Chen et al. [16], the IP rate decreased with a longer interval between salpingectomy and IVF-ET. Their study focused on interstitial pregnancy following IVF-ET, so the interval between salpingectomy and IVF-ET might be relatively controllable. Nevertheless, spontaneous pregnancy constituted 96.6% of cases in our study, so the interval between salpingectomy and subsequent pregnancy was comparatively uncontrollable. Furthermore, Chen et al. only included patients who underwent salpingectomy owing to hydrosalpinx in the study, while 79.3% of cases with salpingectomy in our series were due to ectopic pregnancy. Therefore, more evidences are required to prove this speculation.

Intrauterine operations include induced abortion and hysteroscopic surgery. Consistent with the previous findings [25, 26], parity and previous intrauterine operation did not pose a risk for interstitial pregnancy following ipsilateral salpingectomy as revealed by the multivariable analysis, despite they showed a positive trend of association in the univariable analysis. Pelvic infection is widely accepted risk factors for ectopic pregnancy in the general female population [27]. It is probable that multiparty and previous intrauterine operation might increase the risk of pelvic infection, and finally an increased chance of ectopic pregnancy.

There is no consensus in the literature on how best to manage interstitial pregnancy. Primary treatment of interstitial pregnancy can be surgical or medical. Surgery can be performed by laparoscopy or laparotomy and can be radical or conservative [17, 28]. Although the occurrence of interstitial pregnancy following ipsilateral salpingectomy is rare, it compels us to think whether there is something that can be done to prevent it happening. Cornual resection necessitating myometrial excision may completely avoid the occurrence of interstitial pregnancy after prior salpingectomy, however it will predispose the patient to uterine rupture in subsequent pregnancies, which is most probably a far more dangerous complication than ectopic pregnancy. In 2018 Chen et al. [16] conducted a retrospective, clinical cohort study and found that cornual suture at the time of salpingectomy would help reduce the risk of subsequent interstitial pregnancy after in vitro fertilization. Moreover, tubal occlusion devices may be used to avoid the occurrence of interstitial pregnancy following salpingectomy [29].

Of course, our study has certain inherent limitations. The major one is that it’s a single-center, retrospective, controlled study. Although we tried to minimize selection biases using PSM, the inclusion of confounding factors in matching might not be comprehensive. Nevertheless, devising a multi-center and randomly designed prospective clinical research poses a set of unique challenges due to its low incidence. Second, our results must be interpreted with caution, in particular due to the relatively small sample size and the inability to correctly distinguish the prognostic weight of each matching parameter we used in our PSM analysis. Although we adjusted statistically for differences in age, BMI and previous salpingectomy side to overcome the effect of potential confounders, the fact that women undergoing right salpingectomy were significantly more before PSM(75.9% vs. 51.8%, p < 0.05) as shown in Table 1 requires further consideration. In a retrospective study, Xia et al. [30] analyzed 6186 tubal pregnancy patients, they found that the occurrence of the right-sided tubal pregnancy was more common than the left side. We speculate that interstitial pregnancy following ipsilateral salpingectomy was closely linked with sided dominance of previous salpingectomy, and an additional study would be needed to explore the relation between them. Finally, in addition to interstitial pregnancy after ipsilateral salpingectomy, other clinical parameters have to be analyzed such as interstitial pregnancy after bilateral salpingectomy. It is therefore uncertain if the result also applies to women with the history of bilateral salpingectomy.

## Conclusion

In conclusion, our preliminary finding indicates that there appears to be a significant association between hydrosalpinx and interstitial pregnancy following ipsilateral salpingectomy in subsequent pregnancy. As our results are based on unilateral salpingectomy, the clinical implications of these findings need further elaboration, and larger studies are needed to confirm our results and validate them.

## Data Availability

All data related to this study are available from the corresponding author upon reasonable request.

## Abbreviations

PSM: Propensity score macthing
BMI: Body mass index
ET: Embryo transfer
IVF: In vitro fertilization
